# Composite intestinal adenoma-microcarcinoid in the colon and rectum: a case series and historical review

**DOI:** 10.1186/s13000-017-0665-9

**Published:** 2017-11-07

**Authors:** Mi-Jung Kim, Eun-Jung Lee, Do Sun Kim, Doo Han Lee, Eui Gon Youk, Hyun-Jung Kim

**Affiliations:** 1 Department of Pathology, Daehang hospital, 481-10 BangBae3-dong, Seocho-gu, 137-820 Seoul, Republic of Korea; 2 Department of Surgery, Daehang hospital, 481-10 BangBae3-dong, Seocho-gu, 137-820 Seoul, Republic of Korea; 3 Department of Pathology, University of Inje College of Medicine, Sanggye Paik hospital, Dongil-ro 1342, Nowon-gu, Seoul, Republic of Korea

**Keywords:** Composite intestinal adenoma, Microcarcinoid, Neuroendocrine tumor, Colorectal lesions

## Abstract

**Background:**

Composite intestinal adenoma-microcarcinoid (CIAM) is a rare colorectal lesion that mostly comprises a conventional adenomatous component with a minute proportion of neuroendocrine (NE) component. Although microcarcinoids are well-recognized in the setting of chronic inflammatory disorders of the gastrointestinal tract, large intestinal microcarcinoids associated with intestinal adenoma are exceedingly rare and their clinicopathologic characteristics are yet to be elucidated. This study was performed to clarify their clinicopathologic characteristics and to review the relevant literature.

**Methods:**

In total, 24 cases of CIAM in which tumors were excised endoscopically (*n* = 22) or surgically (*n* = 2) were retrieved from the Department of Pathology, Daehang Hospital. We analyzed their clinicopathologic characteristics and performed immunohistochemical staining for NE markers to determine their endocrine nature.

**Results:**

CIAM usually developed in middle-aged and elderly patients, with a mean age of 62.0 years (range, 44–81 years). Thirteen patients were men and 11 were women, indicating a nearly equal sex ratio. Unlike classic carcinoid tumors, CIAMs occurred mostly in the colon (83.3% of cases), particularly in the proximal colon. Histologically, the microcarcinoid component consisted of low-grade NE cells arranged in small nests, glands or cords interspersed with glandular elements or less frequently resembled squamous morules. There was no expansile nodular or organoid growth pattern, which is typical of carcinoid tumors. The microcarcinoids were 1–20 mm in size (mean size, 4.7 mm) and were mostly situated in the basal lamina propria with no submucosal layer involvement; none showed desmoplastic reaction or increased proliferative activity. Follow-up data (mean, 23.1 months) were available for 18 patients; all patients are alive and well.

**Conclusions:**

To the best of our knowledge, ours is the largest series of patients with CIAM in the English-language literature. Microcarcinoids found in CIAMs appear to show favorable clinical outcomes regardless of their size, likely due to the absence of submucosal extension and/or increased proliferative activity. We recommend avoiding additional radical surgeries in patients who have endoscopically undergone complete CIAM excision unless they exhibit ominous histologic features such as submucosal extension or increased proliferative activity.

## Background

Composite intestinal adenoma-microcarcinoid (CIAM) is a rare colorectal lesion that comprises conventional adenomatous components intermingled with smaller microcarcinoids [1]. Microcarcinoids refer to microscopic aggregates of monotonous cells with neuroendocrine (NE) features that do not form grossly evident masses; they have been well-described in the setting of chronic inflammatory disorders of the gastrointestinal tract, particularly the stomach [2, 3]. Large intestinal microcarcinoids are extremely rare compared to gastric lesions, and have been observed almost exclusively in patients with ulcerative colitis [4–6].

CIAM appears to be a much rarer condition than microcarcinoids that occur in the setting of ulcerative colitis; their NE cells are well-differentiated (WD) and are situated within the basal lamina propria [1, 7, 8]. They have been reported sporadically since they were first described by Pulitzer et al. [1, 7–9]. However, the nature and clinical behavior of CIAMs remain poorly understood. To attain a clearer understanding of this tumor type, we analyzed the clinicopathologic features of 24 new cases of CIAM and also reviewed previous reports of patients with this disease [1, 7–10].

## Methods

### Study sample and histologic evaluation

Twenty-four cases of CIAM were retrieved from the Department of Pathology, Daehang Hospital, between March 2011 and March 2017. Ten were retrospectively collected from pathological data files of patients treated between March 2011 and December 2013, while 14 were identified prospectively between January 2014 and March 2017. All lesions were completely excised either endoscopically (*n* = 22) or surgically (*n* = 2); 1 of the latter lesions was removed by right hemicolectomy because of the presence of a synchronous huge adenoma, and the other was removed via low anterior resection because the physician suspected a malignancy. None of the patients had any history of inflammatory bowel disease (IBD) or familial adenomatous polyposis (FAP).

All specimens were routinely processed, stained with hematoxylin and eosin, and evaluated by a gastrointestinal pathology specialist (M.J.K.). The degree of dysplasia in the epithelial component was assessed according to the architectural complexity, extent of nuclear stratification, and severity of abnormal nuclear morphology, and was classified into low-grade dysplasia (LGD) or high-grade dysplasia (HGD) [11]. Tumors exhibiting lamina propria invasion with no submucosal extension were diagnosed as intramucosal carcinomas.

The sizes of the polyps were measured by the pathologist, while the anatomical locations were identified and classified as the proximal colon (up to the splenic flexure) versus the distal colon/rectum.

The study was performed according to the Declaration of Helsinki, and was approved by the institutional review board at Daehang Hospital (approval number DH17–001). Obtaining additional informed consent for the use of patient samples was not required, as the specimens were coded to protect patient confidentiality.

Patients were assessed for clinicopathologic characteristics including age, sex, and pathology reports. Follow-up data (mean, 23.1 months) were available for 18 patients who underwent endoscopic procedures (*n* = 17) or surgery (*n* = 1) between March 2011 and May 2016. Two patients were lost to follow-up. The remaining samples (*n* = 6) were collected subsequently from patients who had not yet undergone routinely scheduled follow-up visits.

### Immunohistochemical staining

Immunohistochemistry was manually performed by using formalin-fixed, paraffin-embedded blocks. Sections (3 μm) were cut, deparaffinized in xylene, and dehydrated in increasing concentrations of ethanol. Immunohistochemical staining was performed with anti-synaptophysin (clone Z66, 1:100 dilution; Invitrogen, Melbourne, Australia), anti-chromogranin (clone NS55, 1:100 dilution; Invitrogen), and mouse monoclonal anti-Ki-67 (clone 7B11, 1:100 dilution; Invitrogen) after routine microwave antigen retrieval. Negative control samples underwent the same procedure with the omission of the primary antibody. Slides were counterstained with Mayer’s hematoxylin.

Immunoreactivity for synaptophysin and chromogranin was evaluated as positive or negative. Negative protein expression was defined as the complete absence of cytoplasmic staining in the microcarcinoid component in the presence of positive labeling in non-neoplastic internal control cells, while the opposite staining pattern was considered positive expression. Ki-67 immunostaining was performed to determine the proliferative activity and grade of the microcarcinoid NE cells. Nuclear immunostaining at known proliferative locations, such as germinal centers and the basal half of the crypt epithelium, was used as an internal positive control for each sample.

### Statistical analysis

Data analyses were performed using SPSS version 21.0 (SPSS Inc., Chicago, IL, USA). Student’s t-test was used to compare parametric distributions, while the *χ* ^2^ or Fisher’s exact test was used for frequency distributions. A *P*-value <0.05 was considered statistically significant.

## Results

### Patient demographics and tumor characteristics

The patients’ ages ranged between 44 and 81 years (mean, 62.0 years). Samples were obtained from 13 men and 11 women (the male to female ratio was 1.2:1). Among the 24 cases, the majority (*n* = 20; 83.3%) occurred in the colon, the remaining 4 were in the rectum. Sixteen of the 20 CIAMs occurring in the colon (80.0%) affected the proximal colon. The polyp sizes ranged from 5 to 127 mm (mean, 27.2 mm). Follow-up data were available for 18 patients, all of whom are alive and well. The results are summarized in Table 1.

**Table 1:** Clinicopathologic features of the present study

Case	Age (yrs) / Sex	Location of polyp	Procedure	Polyp size (mm)	Histology of glandular component	Initial diagnosis	Size of micro-carcinoid (mm)	SYN	CHR
1	76/F	Ascending colon	ESD	18	IMAC, WD	IMAC, WD	2	+	+
2	68/M	Transverse colon	ESD	25	IMAC, WD	IMAC, WD	4	+	+
3	55/M	Splenic flexure	EMR	27	TA	TA with endocrine cell proliferation	3	+	+
4	65/F	Hepatic flexure	ESD	33	TA	TA	4	+	+
5	49/F	Sigmoid colon	EMR	18	TA	Composite TA and carcinoid tumor	5	+	–
6	74/F	Ascending colon	ESD	23	TVA, HGD	TVA, HGD, with microcarcinoid component	8	+	+
7	55/M	Ascending colon	EMR	10	TA	TA and microcarcinoid	2	+	+
8	47/F	Cecum	EMR	18	TVA	TVA and microcarcinoid	4	+	+
9	61/M	Hepatic flexure	RHC	17	TA	TA and microcarcinoid	2	+	+
10	68/M	Ascending colon	EMR	11	SIAC, WD	Adenocarcinoma, WD, with microcarcinoid component	5	+	+
11	59/M	Splenic flexure	Polypectomy	5	TA	TA and microcarcinoid	1	+	+
12	65/M	Rectum	ESD	20	TVA, HGD	TVA and microcarcinoid	2	+	–
13	78/M	Rectum	ESD	36	TVA, HGD	TVA and microcarcinoid	2	+	–
14	44/M	Sigmoid colon	ESD	17	TA, HGD	TA and microcarcinoid	3	+	+
15	56/F	Ascending colon	ESD	30	TA	TA and microcarcinoid	6	+	+
16	81/F	Rectosigmoid colon	EMR	11	TA	TA and microcarcinoid	2	+	–
17	57/F	Ascending colon	ESD	25	TA, HGD	TA and microcarcinoid	7	+	+
18	46/M	Ascending colon	Polypectomy	20	TVA	TVA and microcarcinoid	2	+	+
19	56/M	Hepatic flexure	Polypectomy	8	TA	TA and microcarcinoid	1	+	–
20	52/M	Rectum	ESD	127	TVA	TVA and microcarcinoid	4	+	–
21	73/M	Transverse colon	Polypectomy	12	TA	TA and microcarcinoid	4	+	+
22	78/F	Rectum	LAR	100	TVA, HGD	TVA and microcarcinoid	20	+	–
23	69/F	Ascending colon	ESD	28	TA	TA and microcarcinoid	13	+	+
24	57/F	Sigmoid colon	EMR	13	TA	TA and microcarcinoid	6	+	+

*CHR* chromogranin, *EMR* endoscopic mucosal resection, *ESD* endoscopic submucosal dissection, *F* female, *HGD* high-grade dysplasia, *IMAC* intramucosal adenocarcinoma, *LAR* low anterior resection, *M* male, *RHC* right hemicolectomy, *SIAC* submucosal invasive adenocarcinoma, *SYN* synaptophysin, *TA* tubular adenoma, *TVA* tubulovillous adenoma, *WD* well-differentiated

### Histologic findings and immunohistochemical staining results

**Fig. 1:**
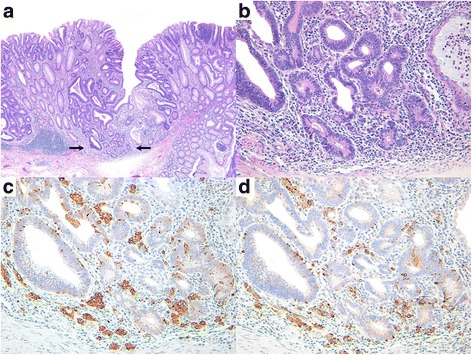
Histological and immunohistochemical findings of composite intestinal adenoma-microcarcinoid. **a** Low power magnification shows adenomatous polyp with hardly recognizable microcarcinoid component (×40). Black arrows indicate microcarcinoid component. **b** High power magnification displays adenomatous glands and neuroendocrine cell nests in the lamina propria, which are connected to each other (×200). **c** Immunohistochemical staining for synaptophysin shows diffuse cytoplasmic reactivity in the microcarcinoid component (×200). **d** The microcarcinoid component is diffusely stained for chromogranin-A on immunostaining (×200)

The microcarcinoid component was most often located in the center of the polyp with no clear demarcation from the glandular component. The microcarcinoids were 1–20 mm in size (mean size, 4.7 mm) and were mostly situated in the basal lamina propria beneath the glandular component (Fig. 1a, b). Only 5 microcarcinoids were focally extended to the muscularis mucosae with no involvement of the submucosal layer, while 1 case involved both the upper and basal portions of the lamina propria.

**Fig. 2:**
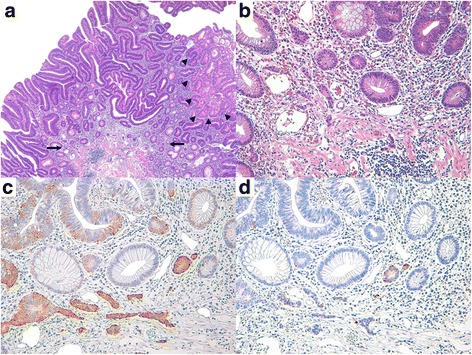
Histological and immunohistochemical features of composite intestinal adenoma-microcarcinoid, mimicking invasive carcinoma. **a** Low power magnification shows adenomatous glands with minute microcarcinoid component (black arrows, ×40). Arrow heads indicate adjacent carcinomatous area. **b** High power magnification reveals endocrine cell nests with angulated shape and infiltrative growth pattern, harboring the potential for misdiagnosis as carcinoma invasion (×200). **c** Immunohistochemical staining for synaptophysin shows diffuse cytoplasmic reactivity in microcarcinoid component (×200). **d** The microcarcinoid component displays focal positivity for chromogranin-A on immunostaining (×200)

**Fig. 3:**
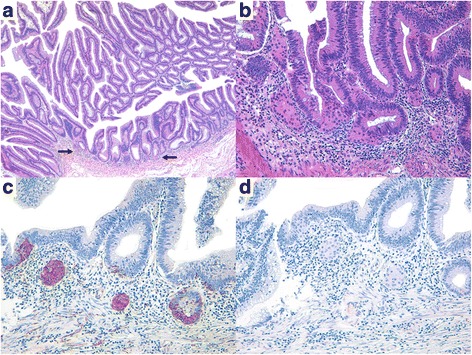
Histological and immunohistochemical features of composite intestinal adenoma-microcarcinoid, mimicking adenoma with squamous metaplasia (squamous morules). **a** Low power magnification shows adenomatous glands with a few eosinophilic cell nests (black arrows) in basal lamina propria (×40). **b** High power magnification shows adenomatous glands and eosinophilic cell nests resembling squamous metaplasia (×200). **c** Immunohistochemical staining for synaptophysin shows diffuse cytoplasmic reactivity in the eosinophilic cells nests, supporting the neuroendocrine differentiation (×200). **d** The neuroendocrine component is negative for chromogranin-A on immunostaining (×200)

Histologically, the NE cells within the microcarcinoid component were arranged in small nests or glands, irregular clusters, and cords with or without connections to the glandular component. Specifically 11 of 24 cases (45.8%) were connected to the glandular component (Fig. 1b). The glandular component was a conventional adenoma with LGD in most cases (86.4%) except for 2 intramucosal and 1 submucosal invasive carcinoma cases. The NE cells were sometimes scattered individually or else resembled infiltrative glands or tumor budding; however, there was no desmoplastic reaction characterized by myofibroblastic proliferation (Fig. 2a, b). Expansile nodular growth patterns or interconnected trabecular and/or lobular structures were not observed in any of the cases. The NE cells had scant to abundant eosinophilic, granular cytoplasm and round central nuclei with stippled or dusty chromatin. Two cases showed endocrine cell aggregates resembling squamous morules or metaplasia (Fig. 3a, b). All microcarcinoids consisted of monotonous cells lacking significant nuclear atypia, mitotic activity, or necrosis; however, some cases showed mild nuclear atypia.

All cases were positive for synaptophysin; moreover, 18 (75.0%) expressed chromogranin-A, which was indicative of their endocrine differentiation (Figs. 1c, d, 2c, d, and 3c, d). The glandular component of the polyps did not show any generalized increase in the expression of NE markers. None of the samples exhibited an increase in the Ki-67 labeling index (all were less than 1%). The results are summarized in Table 1.

## Discussion

Since first described by Pulitzer et al. in 2006, CIAMs have been recognized as a rare intestinal neoplasm consisting of intermingled adenomatous and WD NE components [1]. Unlike other mixed adenoneuroendocrine tumors of the large intestine in which the NE component occupies a substantial proportion of the tumor, the NE component of a CIAM occupies only a minute region of the polyp without disturbing the overall architecture [1, 11]. The NE component found in CIAM differs from classic colorectal neuroendocrine tumors (NETs) in that it does not form a visible nodule and is always accompanied by a glandular neoplasm occupying the majority of the polyp by definition [1]. Additionally, most microcarcinoids in CIAMs are reportedly located in the basal lamina propria, contrary to classic NETs in which the epicenters are located in the submucosa [1, 11]. Therefore, the NE component in CIAMs is always incidentally found during the pathologic examination of adenomatous polyps, while rectal NETs are discovered during routine rectal examinations or endoscopies as submucosal masses or due to the presence of clinical symptoms such as rectal bleeding, pain, or constipation [11]. The majority of colonic NETs are large, with average sizes of 4.9 cm; therefore, they are frequently symptomatic [11].

To date, reports of CIAMs have been sporadic. The largest study was conducted by Salaria et al., who investigated 11 prospectively collected cases over a 7-year period [1, 7–10]. However, their study was limited by the fact that most cases (*n* = 9, 81.8%) were obtained by external consultation; therefore, clinicopathologic data regarding polyp location, procedure type, and immunohistochemical staining results for NE markers were lacking. Another study of 7 CIAM cases by Lin et al. included 5 in the large intestine and 2 in the duodenum [7]. We excluded 2 duodenal cases from our literature review because our study includes CIAMs that occurred only in the colon and rectum. We also excluded 1 of Lin et al.’s remaining 5 cases because the lesion resembled a goblet cell carcinoid based on histologic photographs showing bland-looking glands with prominent goblet cells infiltrating the submucosa [7]. A goblet cell carcinoid is a distinct form of mixed adenoneuroendocrine tumors, which usually shows aggressive biologic behavior despite its bland-looking histology [11, 12]. We also excluded a study published by Estrella et al. because we could not clearly distinguish CIAM cases from adenoma/low-grade NETs [13]; in their study, the majority of cases included in the adenoma/low-grade NET category are presumed to be mixed adenoma/classic NET because a substantial proportion of their cases (40%) invaded the submucosa. Additionally, the authors classified 4 cases arising from the duodenum under the same category as 19 colorectal cases, despite pre-existing evidence indicating that the anatomic site is one of the most important factors that affect the clinical behavior of NETs [11]. Four patients known to have FAP were also included in the adenoma/low-grade NET category, raising our concerns over the pathogenetic heterogeneity of their cases, which can potentially affect the analysis of clinical outcomes. After excluding the above-mentioned cases, we summarized the clinicopathologic data of 21 previously reported CIAMs in Table 2. In our present study, we enrolled and analyzed CIAM cases that only occurred in the colon and rectum, and did not include any patients with a history of IBD or FAP to minimize the genetic and pathogenetic heterogeneity of the investigated cases (Table 1).

**Table 2:** Clinicopathologic features of the previous studies

Case	Reference	Age (yrs) / Sex	Location of polyp	Procedure	Polyp size (mm)	Histology of glandular component	Initial diagnosis	Size of micro-carcinoid (mm)	SYN	CHR
1	Lyda et al., 1988 [10]	80/M	Ascending colon	Polypectomy followed by RHC	30	TVA, HGD	TVA and carcinoid tumor	NS	+	NS
2	Pulitzer et al., 2006 [1]	77/F	Cecum	Polypectomy followed by RHC	<10	TA, HGD	TA with HGD and endocrine neoplasia of UMP	NS	+	+
3	Pulitzer et al., 2006 [1]	77/M	Cecum	Polypectomy	13	TA	TA with squamous metaplasia	NS	+	–
4	Pulitzer et al., 2006 [1]	77/F	Cecum	Polypectomy followed by RHC	20	SIAC	TVA, HGD	NS	+	+
5	Pulitzer et al., 2006 [1]	62/F	Descending colon	Polypectomy	5	TA	TA with microcarcinoid	NS	+	+
6	Lin et al., 2012 [7]	51/M	Rectum	Polypectomy followed by proctosigmoidectomy	20	TVA	TVA with microcarcinoid	1	+	+
7	Lin et al., 2012 [7]	59/M	Rectum	Polypectomy followed by proctosigmoidectomy	13	Adenoma	Adenocarcinoma	1	+	+
8	Lin et al., 2012 [7]	66/F	Cecum	Polypectomy followed by RHC	15	TA	TA and microcarcinoid	1	NS	NS
9	Lin et al., 2012 [7]	56/M	Sigmoid colon	Polypectomy followed by transanal excision	25	TVA	TVA and microcarcinoid	1	NS	NS
10	Salaria et al., 2013 [8]	55/M	Right colon	NS	53	TA	Suspicion of invasive carcinoma	2	+	NS
11	Salaria et al., 2013 [8]	54/F	Transverse colon	NS	14	TA, HGD	TA, HGD	1	NS	NS
12	Salaria et al., 2013 [8]	81/F	Left colon	NS	12	TVA, HGD	TVA, HGD	3	+	–
13	Salaria et al., 2013 [8]	28/F	Right colon	NS	34	TVA, HGD	TVA, HGD, with squamous morules	7	+	–
14	Salaria et al., 2013 [8]	82/M	Right colon	NS	15	TVA	Suspicion of invasive carcinoma	5	+	–
15	Salaria et al., 2013 [8]	72/F	NS	NS	15	TVA	NS	5	NS	NS
16	Salaria et al., 2013 [8]	60/F	Right colon	NS	7	TVA, HGD	TVA, HGD	3	–	–
17	Salaria et al., 2013 [8]	55/M	Left colon	NS	12	TA	TA with squamous morules	7	NS	NS
18	Salaria et al., 2013 [8]	48/M	Left colon	NS	15	TVA	TVA with squamous morules	4	+	–
19	Salaria et al., 2013 [8]	62/M	Left colon	NS	25	TVA	TVA with squamous morules	4	NS	NS
20	Salaria et al., 2013 [8]	51/F	Left colon	NS	30	TVA	TVA with squamous morules	2	NS	–
21	Thosani et al., 2014 [9]	73/M	Hepatic flexure	NS	NS	TVA	NS	NS	+	–

*CHR* chromogranin, *F* female, *HGD* high-grade dysplasia, *M* male, *NS* not specified, *RHC* right hemicolectomy *SIAC* submucosal invasive adenocarcinoma, *SYN* synaptophysin, *TA* tubular adenoma, *TVA* tubulovillous adenoma, *UMP* uncertain malignant potential, WD well-differentiated

Herein, we summarized the clinicopathologic findings of a total of 45 CIAM cases, including results from previous studies as well as our own. There were 24 men and 21 women, indicating a nearly equal sex ratio (male:female = 1.1:1). The patients’ ages ranged from 28 to 82 years, with a mean age of 62.6 years. Notably, the mean age among our 24 patients (62.0 years) did not significantly differ from the 21 patients in the previous studies (63.1 years, *p* = 0.767). Approximately two-thirds (*n* = 29, 64.5%) of the microcarcinoid components were accompanied by adenoma with LGD (17 tubular, 11 tubulovillous, and 1 unspecified), 12 by adenoma with HGD (4 tubular, 8 tubulovillous), 2 by intramucosal carcinoma arising in tubulovillous adenoma, and 2 by submucosal invasive adenocarcinoma. Therefore, we suggest that the terminology of “CIAM” might be misleading because microcarcinoids can be associated with glandular lesions that exhibit various histologic degrees of dysplasia.

Contrary to classic NET, CIAMs tended to occur in the colon according to data from previous studies as well as ours, which indicated that most lesions (38 of 44, 86.4%) were located in the colon except for 6 (13.6%) that occurred in the rectum and 1 that had an unknown location. In particular, more than half of the polyps were located in the proximal colon (27 of 44 cases, 61.4%), with the most frequent site being the ascending colon (9 of 44 cases, 20.5%). The size of the polyps ranged from 5 to 127 mm, with a mean size of 23.8 mm. There was no significant difference in polyp size between our study and previous findings (27.2 mm vs. 19.6 mm, respectively; *p* = 0.277). The microcarcinoid component was confined to the mucosa with (22 of 40 cases) or without (18 of 40 cases) a connection to the glandular component. The mean size of the microcarcinoid component was 4.7 mm, and did not differ significantly in our patients compared to those in previous studies (4.7 mm vs. 3.1 mm, respectively; *p* = 0.201).

In our study, all lesions were completely removed either by endoscopic procedures (*n* = 22) or surgical resection (*n* = 2), and no subsequent surgeries were required. This is inconsistent with the results of previous studies in which most patients underwent subsequent surgery (*n* = 7, 77.8%) because of incomplete or partial prior polypectomy (*n* = 9). Moreover, removal of the majority of samples (83.3%) by endoscopic mucosal resection (EMR, *n* = 7), endoscopic submucosal dissection (ESD, *n* = 11), and surgery (*n* = 2) in our study facilitated the procurement of well-oriented tissue sections perpendicular to the basal lamina and muscularis mucosae; this assisted us in collecting the largest series of CIAM samples. A microcarcinoid component cannot be detected in poorly-oriented tissue samples such as small polypectomy specimen because it is mainly located in the basal lamina propria. Nevertheless, the prevalence rate of CIAM appears to be extremely low based on our estimates. We prospectively collected 13 CIAM cases from among 40,939 patients who underwent endoscopic procedures including polypectomy, EMR, and ESD between January 2014 and March 2017.

So far, the natural history as well as the pathogenetic mechanism of colorectal microcarcinoids have not been fully elucidated because of their rarity, which in turn may partly be due to their under-recognition. As a result, microcarcinoids occurring in the colon and rectum have remained an ambiguous entity denoting small-sized NE lesions in many instances. According to the definition of microcarcinoid described by Pulitzer et al., most previously reported microcarcinoids occurring in ulcerative colitis patients appear to correspond to small-sized classic NETs [1]. As for the stomach, most enterochromaffin-like cell NETs arise in patients with chronic atrophic gastritis or multiple endocrine neoplasia type 1-Zollinger-Ellison syndrome through a sequence of hyperplasia-dysplasia-neoplasia, where growth patterns as well as endocrine cell sizes are known to be important for the classification of such lesions [2, 3, 11]. A gastric NE lesion is classified as a microcarcinoid when the nodule is greater than 0.5 mm but less than 5 mm in size, or if it invades the submucosa; lesions less than 0.5 mm and confined to the mucosa are designated as carcinoma in situ/dysplasia [11]. However, to our knowledge, such size-based criteria have not yet been defined in colorectal microcarcinoids. Based on our findings, the clinical outcomes of colorectal microcarcinoids appear to be quite favorable regardless of their sizes, likely because none of the patients showed submucosal invasion and/or increased proliferative activity. Considering that none of the 45 CIAM patients showed recurrence or metastasis after endoscopic or surgical treatment, even in lesions larger than or equal to 5 mm (8 of 39 cases, 20.5%), the 5 mm-size cutoff appears to be meaningless. Therefore, we posit that the absence of submucosal invasiveness and/or proliferative activity of the NE cells, and not their size, explain the favorable biologic behavior of microcarcinoids.

It is also worth considering whether or not colorectal microcarcinoids are neoplastic lesions and how best to define them. We suggest that it is premature to define colorectal microcarcinoids as neoplastic lesions, particularly when these lesions are confined to the mucosa with no obvious signs of proliferative activity. Indeed, 7 of 9 patients (77.8%) in previous studies underwent additional surgery after polypectomy for fear of residual lesions and possible ominous outcome. We suggest that intramucosal WD NE lesions in the colorectum should be distinguished from classic small-sized or microscopic NETs.

From a pathologist’s perspective of view, awareness of microcarcinoids is critical; however, it is also important to avoid over-interpretation while not overlooking or under-recognizing such lesions. Pathologists can misinterpret microcarcinoids as high-grade lesions such as invasive components of associated glandular lesions, particularly when microcarcinoids show infiltrative or single-cell patterns, or else can consider them small-sized classic NET. In the former case, identifying desmoplastic reactions that appear as myofibroblastic proliferation as well as checking for NE differentiation are important. According to our analysis, the most common mistake was to overlook the microcarcinoid component (7 of 43 cases, 16.3%), followed by misdiagnosis of the microcarcinoid as a squamous metaplasia (squamous morules) (6 cases, 14.0%) or invasive glandular component (3 cases, 7.0%) [1, 7, 8]. To that point, a CIAM reported by Lyda et al. was diagnosed as a composite adenoma-carcinoid tumor [10].

## Conclusions

To our knowledge, we have reported the largest series of colorectal CIAM. Clinically, CIAM tends to develop in middle-aged to elderly patients and manifests as a colorectal polyp that is usually located in the proximal colon. Microcarcinoids found in CIAM exhibits 2 histologic patterns: they are more commonly observed as WD NE cells arranged in small clusters, glands, or cords interspersed with glandular elements; and are less commonly observed as cell aggregates resembling squamous morules. Microcarcinoids found in CIAMs appear to have favorable clinical outcomes, likely because they are not accompanied by submucosal extension and/or increased proliferative activity. We recommend avoiding further radical surgeries in patients with CIAM that was completely removed endoscopically unless they show submucosal extension or increased proliferative activity.

## Abbreviations

CIAM: Composite intestinal adenoma-microcarcinoid
EMR: Endoscopic mucosal resection
ESD: Endoscopic submucosal dissection
FAP: Familial adenomatous polyposis
HGD: High-grade dysplasia
IBD: Inflammatory bowel disease
LAR: Low anterior resection
LGD: Low-grade dysplasia
NE: Neuroendocrine
NET: Neuroendocrine tumor
TA: Tubular adenoma
TVA: Tubulovillous adenoma
WD: Well-differentiated
